# Novel Insights for Metabiotics Production by Using Artisanal Probiotic Cultures

**DOI:** 10.3390/microorganisms9112184

**Published:** 2021-10-20

**Authors:** Marina Pihurov, Bogdan Păcularu-Burada, Mihaela Cotârleţ, Mihaela Aida Vasile, Gabriela Elena Bahrim

**Affiliations:** Faculty of Food Science and Engineering, Dunarea de Jos University of Galati, Domneasca Street No. 111, 800201 Galati, Romania; marina.pihurov@ugal.ro (M.P.); bogdan.pacularu@ugal.ro (B.P.-B.); mihaela.cotarlet@ugal.ro (M.C.); aida.vasile@ugal.ro (M.A.V.)

**Keywords:** milk kefir grains, water kefir grains, kombucha, probiotics, postbiotics, paraprobiotics, metabiotics

## Abstract

Wild probiotic consortia of microorganisms (bacteria and yeasts) associated in the artisanal cultures’ microbiota (milk kefir grains, water kefir grains and kombucha) are considered valuable promoters for metabiotics (prebiotics, probiotics, postbiotics and paraprobiotics) production. The beneficial effects of the fermented products obtained with the artisanal cultures on human well-being are described by centuries and the interest for them is continuously increasing. The wild origin and microbial diversity of these above-mentioned consortia give them extraordinary protection capacity against microbiological contaminants in unusual physico-chemical conditions and unique fermentative behaviour. This review summarizes the state of the art for the wild artisanal cultures (milk and water kefir grains, respectively, kombucha—SCOBY), their symbiotic functionality, and the ability to ferment unconventional substrates in order to obtain valuable bioactive compounds with in vitro and in vivo beneficial functional properties. Due to the necessity of the bioactives production and their use as metabiotics in the modern consumer’s life, artisanal cultures are the perfect sources able to biosynthesize complex functional metabolites (bioactive peptides, antimicrobials, polysaccharides, enzymes, vitamins, cell wall components). Depending on the purposes of the biotechnological fermentation processes, artisanal cultures can be used as starters on different substrates. Current studies show that the microbial synergy between bacteria—yeast and/or bacteria—offers new perspectives to develop functional products (food, feeds, and ingredients) with a great impact on life quality.

## 1. Introduction

One of the most effective public health methods for preventing infectious illnesses is to fortify immunity via functional nutrition [1]. Gut bacteria can affect viral infection by influencing the numbers of regulatory T-cells which development is specifically influenced by the short-chain fatty acids (SCFAs) resulting from the fermentation of dietary fibres [2]. As a result, the synergism between food and microbiota plays an important role in avoiding immune-mediated diseases and viral infections [3]. The epidemic encouraged consumers to be interested in immune-boosting functional foods, which are based on bioactive components for sustaining physical and psychological health [4]. Functional food and ingredients have additional benefits on human physiology and metabolic functions throughout supplying with essential nutritional needs of the body, thereby aiding in disease prevention and living a healthy life and these are recommended to be used in diets [5]. On the global health system, bioactive compounds help to reduce obesity, decrease the risk of cancer, strengthen the immune system, sustain bowel well-being, optimize cardiovascular and urinary system, eye functions, and provide anti-inflammatory, antibacterial, and antiviral properties and they are also very adaptive to the dynamic human microbiome [6,7]. Short peptides obtained by the simulated gastrointestinal digestion of soybean protein extracts were evaluated for antioxidative activity by an intracellular test [8], while peptide fragments were obtained from yellow fin tuna muscle by simulated gastrointestinal digestion, and their antimicrobial activity towards Gram-positive and Gram-negative bacteria was investigated [9].

The mammalian gut is represented by four dominant bacterial phyla *Firmicutes*, *Bacteroidetes*, *Actinobacteria*, and *Proteobacteria* [10] with 500 to 1000 different bacterial species. The interaction between the microbiota and the host organism has a great impact on the communication signals and functionality, it is also influenced by external factors. The microorganisms produce a variety of signal molecules such as peptides, amino acids, SCFAs and gaseous compounds. They respond to the signals from their hosts, in this way the connection between the nervous system of the host and its organism being kept through the gut-brain axis [11].

For maintaining a good function of gut microbiota the functional nutrition embraces a large number of essential elements as bioactive promoters such as fat-soluble and water-soluble vitamins (A, D, E, B1, B2, B6, B12, B9, C, PP) [12] with a synergistic role with lipoic and orotic acids, essential amino acids, polyunsaturated fatty acids (omega-3, omega-6), phospholipids, antioxidants (vitamins C, E, carotenoids), macroelements and microelements (calcium, magnesium, potassium, iodine, selenium, iron, zinc), prebiotics (dietary fibres, carbon-containing compounds of microbial and non-microbial origin, plant and microbial polysaccharides), probiotics (microorganisms), synbiotics (probiotics and prebiotics), complex mixtures (synbiotics and functional ingredients, such as vitamins and minerals premixes, phenol-based and other plant compounds), and metabiotics (all metabolite products, cells components and viable or/and nonviable cells of the probiotic cultures) [13].

The best known bioactives are probiotics, which are useful in maintaining a healthy digestive tract and increasing immunity. Probiotics are classified into many classes based on the active principle that they contain, as following: (i) autoprobiotics, the active principle is that strains belong to the natural microbiota; (ii) homobiotics, the active principle is that strains isolated from a specific species of animal; (iii) heteroprobiotics are probiotic bacteria that are recommended to be used for animals and humans without regard for their origin [14].

According to a global assessment of the market for immune-stimulating foods (functional food and ingredients) the worldwide market for this type of food is predicted to expand from 830 billion dollars per year to 1 trillion dollars in 2023 [15]. However, probiotics require complex growth conditions, high maintenance cost, susceptibility to the gastrointestinal environment, pathogenic gene transfer, cell lysis at extreme acidic pH, widespread antibiotic resistance, and lower bacterial viability due to the lack of spore formation, along with the biological risks associated with long and uncontrolled usage of classical probiotics [16]. In this context, to sustain the complex and varied microbial consortium of the gut which contains various bacteria, archaea, viruses, and fungi, a new concept of probiotic cell components, metabolites, and signal molecules which is a more stable and efficient alternative for probiotics is developed. “Hidden soldiers” for fortifying the entire army of the immune system of the host has been designated as “metabiotics” which cover prebiotics, probiotics (nutribiotics, pharmacobiotics), synbiotics, paraprobiotics and postbiotics [17].

A source for the concept of metabiotics that is currently not being exploited at full capacity is represented by the artisanal cultures, also called wild consortia of microorganisms, such as milk kefir grains, water kefir grains and kombucha. The connection between metabiotic-artisanal cultures will be analysed from different perspectives.

## 2. Definitions and New Connections between Metabiotics and Artisanal Cultures

*Probiotics. Prebiotics. Synbiotics.* A functional combination of probiotics (live microorganisms with health effects) and prebiotics (nonabsorbable polysaccharides/oligosaccharides) represents a synbiotic, whereas the combination of two or more probiotic strains without prebiotics is defined as a symbiotic. The well-studied and classified prebiotics are fructans (fructooligosaccharides, oligofructose, and inulin), galactans (galactooligosaccharides) and mannanoligosaccharide, while lignin, cellulose, and hemicellulose, resistant starch, polydextrose, xylooligosaccharides, pectin, and human milk oligosaccharides are considered prebiotic candidates [18,19].

The synergistic interaction of prebiotics and probiotics provide a positive effect to the physiological functions of the body, thus indirectly influencing and improving health. By fermentation in the intestine, prebiotic fibres produce short-chain fatty acids and gut hormones that influence the intestinal viscosity, nutrient absorption and stimulate the growth of the benefice gut microorganisms [20]. Furthermore, lectin systems of symbiotic/probiotic microorganisms that recognize the glycoconjugates is another example of specific mechanisms that contribute to the well-being estate of humans and animals [21,22,23].

*Postbiotics.* Postbiotics are water-soluble metabolites or cells’ components resulting after lysis with a molecular weight between 10–100 kDa [24]. Cell-free supernatants that contain postbiotics have a high concentration of SCFAs, amino acids, proteins and peptides, organic acids, enzymes, vitamins, or biosurfactants. Postbiotics can be safely dosed as a food supplement to increase the shelf-life of the product [25]. Postbiotic compounds have high antimicrobial activity and resistance at high temperatures [26].

*Paraprobiotics.* The studies of the functional properties of non-viable microbial probiotic cells started in 2004 [27,28,29]. Paraprobiotics (also called ghost or killed probiotics) are inactivated microbial cells, cell lysates or microbial fractions originating from probiotic microorganisms (i.e., teichoic acids, cell surface proteins, peptidoglycans, chitin, pili, fimbriae surface protruding molecules, the bacterial DNA) that can provide physiological benefits to the host by supplying additional bioactivity. Paraprobiotics are considered to be a safer alternative to probiotics for children born prematurely [30,31], also with anti-inflammatory and positive immune responses for animals and humans.

*Nutribiotics and pharmabiotics.* Probiotic microorganisms (such as *Lactobacillus acidophilus, Bifidobacterum bifidum,* *Streptococcus thermophilus*) with nutritional functions which produce and sustain the vitamin balance of the human host can be named nutribiotics. Their action is based on the production of specific metabolites such as thiamine (B1), riboflavin (B2), niacin (B3), pantothenic acid (B5), pyridoxine (B6), biotin (B7), folate (B9-11), cobalamin (B12), and vitamin K, conjugated linoleic acid (CLA). Nutribiotics can be used for nutraceutical diets and for fortifying foods (especially fermented foods). Probiotics with pharmacological functions for prevention or treating diseases (infectious diarrhea, inflammatory bowel diseases, obesity and diabetes) are named pharmabiotics [32]. Strains like *L. salivarius* and *Lactococcus lactis* are an example of pharmabiotics due to their propriety to produce antimicrobial peptides and proteins such as bacteriocins which are known as pathogens inhibitors and treatment for malignant cancers [33,34].

*Metabiotics.* The novel concept of metabiotics is currently promoted in the new fields of biotics production and use with impact in promoting a healthy life for humans and animals. The metabiotics include biologically active metabolites, signalling molecules and cells (dead microbial cells and their fragments) mainly responsible for in vitro and in vivo effects. The term metabiotic contains the Greek prefix *meta*- (change, transformation), referring to the metabiotic’ ability to initiate a large number of hormonal and neurochemical processes [35]. Metabiotics are microbial low molecular weight compounds (mLMWC) that can be used as remedies, bioactive food additives or as enriching microbial ingredients of functional foods for reconditioning the gut microbiota and health restoring, especially for some individuals. Metabiotics can be well-dosed, at the same time being safe with a long shelf-life. Due to these properties, the period of storage for some food products can be extended when the right amount of metabiotics is used [16,36].

**Figure 1:**
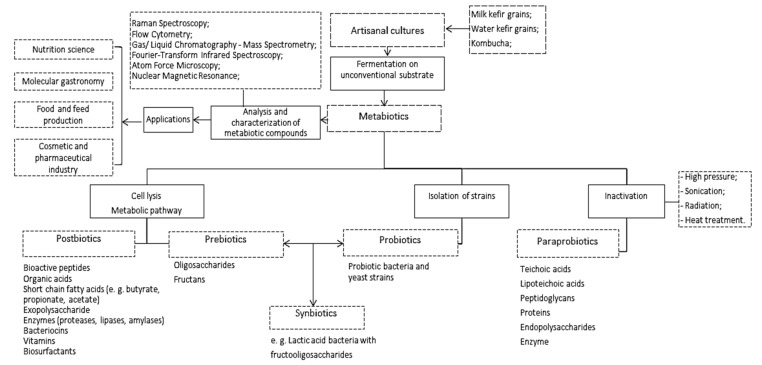
Artisanal cultures as valuable multi-promoters of metabiotics.

*Connections between metabiotics and artisanal cultures.* Artisanal cultures are symbiotic consortia of bacteria and yeasts embedded in a natural complex matrix of exopolysaccharides, proteins, lipids, sugars, amino acids, and nucleic acids. The best known, studied, and utilized artisanal cultures are milk kefir grains, kombucha and water kefir grains. The obtained fermented products are considered probiotic drinks or food beverages because of the implication of probiotic strains from the above-mentioned microbial consortia. Based on the complex metabolic activity of these microorganisms, a large spectrum of compounds with complex function can be obtained (Figure 1).

In artisanal cultures, the wild microorganisms of the consortium are more actively because act in synergism and these microorganisms are more resistant to different intrinsic (e.g., nutrient content, moisture content, pH), extrinsic (e.g., temperature, relative humidity, gases), and biological conditions (e.g., presence, dynamics, and type of interaction of microorganisms) of the ecosystems. In these artisanal cultures, cells are naturally encapsulated in the matrices made of proteins, lipids, and sugars that act as immobilization structures, thus offering protection of the cells. The microorganisms within these artisanal consortia have complex nutritional requirements. Therefore, they can metabolise nutrients from different unconventional substrates which allow them to be used in efficient biotechnological processes in order to obtain bioactive compounds with technological and functional properties with applications in food and feed, bioingredients, pharmaceuticals and cosmeceuticals production [37,38,39]. The symbiotic community based on the water or milk kefir grains and kombucha are complex probiotics and they are used in a large area of fermented products [40].

## 3. Microbial Diversity and the Synergistic Relationships of the Artisanal Cultures

Establishing the microbiota composition of the artisanal consortia is difficult because many autochthonous strains grow hard in controlled conditions. For that, the use of advanced techniques for taxonomic analysis is necessary. The diversity of the microbiota is influenced by the geographical areas or by the environments from which these cultures are naturally formed and used. Furthermore, the continuous study on the microbial taxonomy requires their reclassification and re-nomination at genus or species level [41,42]. The genus *Lactobacillus* is predominant in milk and water kefir grains, the lactic acid bacteria strains that belong to this genus being rarely found in kombucha. *Lactobacillus* comprises 261 species, all of which are phenotypically, ecologically, and genotypically diverse. Based on the species’ core genome phylogeny, pairwise average amino acid identity (conserved), clade-specific signature genes, physiological requirements, *Lactobacillus* genus was reclassified and the *Lactobacillaceae* family was expanded including all the genera previously classified as belonging to the *Lactobacillaceae* and *Leuconostocaceae* families [43].

In this way, for the genus *Lactobacillus*, the new taxonomy’s classification was done as it is presented in Table 1 (adapted from Zheng et al. [43]).

### 3.1. Water Kefir Grains

**Figure 2:**
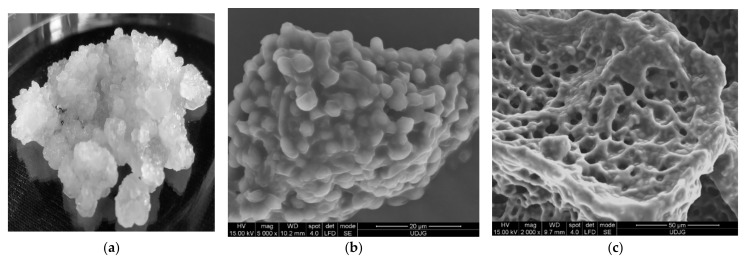
Water kefir grains (**a**) and Scanning Electron Microscopy (SEM) micrographs of water kefir grains at the surface at ×5000 magnification (**b**), at the surface at ×2000 magnification (**c**).

Water kefir is an artisanal fermented product as a low-acid, straw-coloured, and mildly alcoholic non-dairy fermented beverage, produced by a mixture of water, sucrose, dried figs, and other ingredients, inoculated with water kefir grains. A yellowish fermented beverage with a fruity, astringent flavour is obtained after 2–4 days of fermentation at room temperature. Water kefir is consumed all over the world, but the origin of the grains is still uncertain. Other names for the water kefir grains depend on the geographical area, some examples being ginger beer plants, Tibicos, Tibi grains, California bees, African bees, ale nuts, balm of Gilead, Bèbées, Japanese beer seeds, and sugary kefir grains. Transparent water kefir grains (Figure 2a) comprise symbiotic wild strains of lactic acid bacteria (LAB), acetic acid bacteria (AAB) and yeasts used in the production of water kefir. The cells are encapsulated in a glucose polymer—dextran, formed by linear α-D-1,6-and α-1,3-glycosidic bonds, which form transparent, mucilaginous exopolysaccharides matrix, like jelly crystals [44].

**Figure 3:**
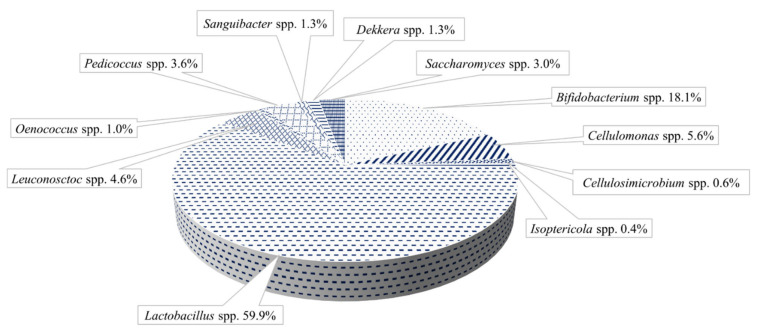
Water kefir grain repartition (adapted from Verce et al. [48]).

The polymer matrix (Figure 2b,c) assures the support and protection of cells and permits the active diffusion of the nutrients and metabolites produced by the immobilized cells. The water kefir grains’ microbiota contains different genera of LAB and AAB, mainly *Lactobacillus* spp., *Leuconostoc* spp. *Bifidobacterium psychraerophilum, B. crudilactis* and *Acetobacter* spp., yeast strains such as *Saccharomyces* spp., *Lanchancea* spp., *Hanseniaospora* spp. and *Zygotorulaspora* spp. genera, associated in a very stable and functional active consortium (Figure 3) [40,45,46,47].

Gulitz et al., reported that *Lactobacillus* spp. population ranged between 82.1–72.1% (especially the species *L. hordei* and *L. nagelii*). In addition, *Leuconostoc mesenteroides* and *Leuc. citreum* were presented in three different origin water kefir grains. The production of exopolysaccharides was evaluated for some species of *Lactobacillus casei, L. hordei, L. nagelii, L. hilgardii* and *Leuc. mesenteroides*, *Lactobacillus* spp. and *Acetobacter* spp. strains were identified in cluster analyses of RAPD-PCR. Furthermore, 26S rDNA sequences and FT-IR (Fourier-transform infrared spectroscopy) showed the presence of *Saccharomyces cerevisiae, Lachancea fermentati, Hanseniaospora valbyensis* and *Zygotorulaspora florentina* species [49]. *Liquorilactobacillus hordei* (former *Lactobacillus hordei*) is described as very adaptive bacteria from the water kefir consortium which produced dextran with high molecular weight [50]. Other researchers observed that, by cultivation of the water kefir grains in a fermentation substrate made of pitaya pulp allowed the bacterial strains like *Liquorilactobacillus satsumensis* (49%), *Oenococcus kitaharae* (29.99%), *Oenococcus oeni* (14.43%) to dominate the microbial consortium [49].

### 3.2. Milk Kefir Grains

**Figure 4:**
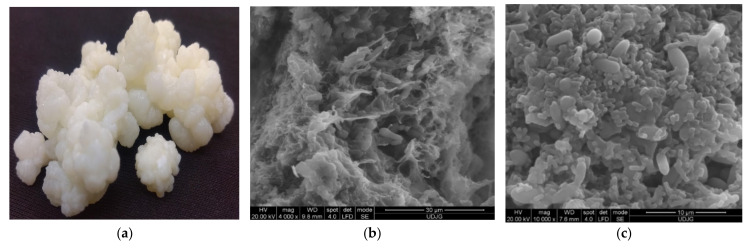
Milk kefir grains (**a**) and Scanning Electron Microscopy (SEM) micrographs of milk kefir grains in the section at ×4000 magnification (**b**), at the surface at ×10,000 magnification (**c**).

For centuries, milk kefir grains have been used as an artisanal starter culture for traditional milk kefir production because of their non-standardized microbial composition that enhances the sensorial, nutritional, and functional traits of the final products. Kefir is a dairy fermented, slightly sour, and creamy with acidic taste product that originated from the Caucasus Mountains. Due to its nutritious content and health benefits, it is a known drink in the world, from Japan to Europe. Some health benefits associated with milk kefirs’ consumption are: decreasing the allergic reactions of lactose-intolerant consumers, antimicrobial (against *Bacillus subtilis*, *Staphylococcus aureus*, *Enterococcus faecalis*, *Escherichia coli*, *Salmonella enteritidis*) [51], antitumoral, antioxidant, antimutagenic, and antiapoptotic effects. Milk kefir grains are elastic, irregular, gelatinous, ivory, or white in colour, from 1 to 5 cm in diameter of cauliflower structure (Figure 4a). The grains contain 44% fat, 12% ash, 45% mucopolysaccharide, 34% total protein (27% insoluble, 1–6% soluble, and 5–6% free amino acids), vitamins B and K, tryptophan, Ca, P, and Mg. Kefir grains consist of a polysaccharide matrix (Figure 4b), named kefiran which links bacteria and yeasts (Figure 4c) [52,53,54]. In Figure 4b,c, the attached bacteria (rods/cocci shapes) and yeasts (spherical/lemon-shaped cells) to the matrix can be observed.

The species that occur in the milk kefir grains depends on the geographical origin of the grain or the cultivation protocols and the fermentation substrates may be the cause for such differences [55]. The fermentation substrate directly impacts the microbiota by quantitative variation of species.

**Figure 5:**
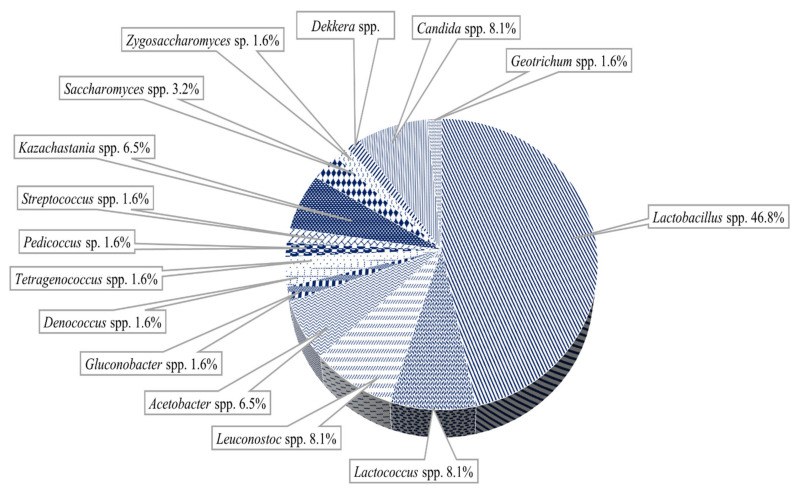
Predominant microorganisms in the milk kefir grains microbiota (adapted from Bengoa et al. [56]).

Milk kefir grains contain homofermentative and heterofermentative strains of LAB (Figure 5) belonging to *Lactobacillus* spp. which represent dominantly genus, 65–80% or 99.42–99.79% such as *Lentilactobacillus buchneri* (formerly *Lactobacillus buchneri*)*, L. kefiranofaciens, L. helveticus, L. kefiri, L. ultunensis, L. gigeriorum, L. apis, Levilactobacillus brevis* (formerly *L. brevis)*, *L. acidophilus*, *Lacticaseibacillus casei* (formerly *L. casei*), *L. helveticus*, *L. delbrueckii*). The lactococci (20–35%), such as *Lactococcus* spp., *(Lactococcus lactis* ssp. *thermophilus*.) *Leuconostoc* spp. *(Leuc. mesenteroides* and *Leuc. cremoris*), *Streptococcus salivarius* and *S. thermophilus* were identified.

Yeasts (0.58–0.21%) such as *Kluyveromyces* spp., *Torula* spp., *Candida* spp. *Torulopsis* spp. and *Saccharomyces* spp. are also present.

Some acetic acid bacteria: *Acidocella aluminiidurans*, *Acetobacter* spp. (*Acetobacter pasteurianus*, *Acetobacter orleanensis*), *Gluconobacter* spp. *Gluconobacter morbifer*, *Gluconobacter frateurii* [57,58,59,60].

LAB strains of *Lactococcus lactis*, *Leuc. mesenteroides*, *Lactiplantibacillus plantarum* (formerly *Lactobacillus plantarum*), *Latilactobacillus sakei* (formerly *Lactobacillus sakei*) were identified in some milk kefir grain samples from different Russian samples. Hence, the kefir grains from Stavropol were dominated by the *L. delbrueckii* strains, whereas strains of *Leuc. gelidum*, *Leuc. pseudomesenteroides*, *L. kefiri* and *Lactococcus lactis* seemed to be the most abundant species within the milk kefir grains consortium [61,62].

In addition to bacteria and yeast, the microbial consortium of milk kefir grains contains phages, also known as bacteriophages. The low level of phages increase the fermented product preservation and food safety assurance with health-supporting properties. Phages co-exist with bacteria and achieve equilibrium between species in the consortium [63]. On the other hand, the presence of a large quantity of phage can damage the quality of the fermented product and represent a biological risk for the product. Bacteriophages of the *Siphoviridae* family, morphotype B1, (represent 60% of the known *Lactobacillus* phages) are specific for milk kefir grains and the fermented product. This type of phage has an icosahedral capsid of 40 to 76 nm in diameter and a tail of 116 to 500 nm in length [64,65].

### 3.3. Kombucha

**Figure 6:**
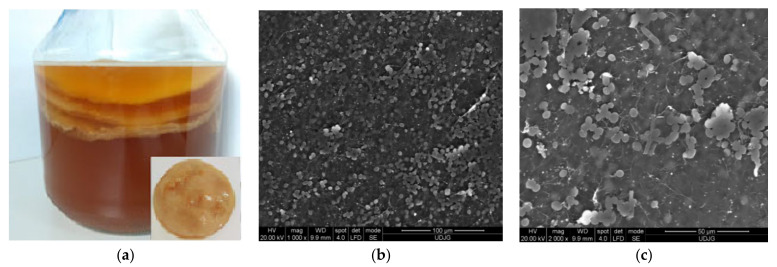
Kombucha biofilm (**a**); SEM images of kombucha biofilm (**b**) at ×1000 magnification and (**c**) at ×2000 magnification.

Kombucha, also known as *Medusomices gisevii*, originated from Manchuria, China. The popularity of this probiotic functional drink, Kombucha tea, lead to the provided essential nutrients, vitamins, metabolites, proteins, and fibres by the fermentation process of 7–10 days of SCOBY (Symbiotic Culture of Bacterium and Yeast) biofilm in sugary tea. During the fermentation of polysaccharides to simple carbohydrates, carboxylic acid and carbonic gas resulted which increase the consumers’ preferences [66]. Furthermore, the SCOBY is used as a starter for a new fermentation. The bacteria-produced cellulose filament that grows at the surface of the fermentation liquid/substrate accumulates in a biofilm (Figure 6a). For each subsequent fermentation, this biofilm grows further to create several pancake-like layers bounded by filaments [67] formed by bacteria and yeasts which can be observed in Figure 6b,c.

The beverage is produced as a result of the mutual interactions of the microorganisms from a stable consortium. Often, AAB and yeast strains that ferment the sugared tea liquor are responsible for the functional metabolites produced, but sometimes LAB strains are involved in the fermentation process, resulting in acetic, gluconic, glucuronic, ethanoic acids. The substrate of fermentation, such as black tea or green tea infusions, can provide the necessary nutrients for the proper development of specific microorganisms. Yeast invertase converts sucrose into glucose and fructose that are further used for the alcoholic fermentation and ends with ethanol production. Through oxidative metabolism, AAB converts glucose to gluconic acid and ethanol to acetic acid, thus proving the symbiotic relationships that contribute to the enhancement of kombucha’s sensorial and antimicrobial features [68].

At different stages of fermentation, the composition of the kombucha microbiome can vary [69] and it is produced by *Rhodospirillales* cluster, *Firmicutes, Proteobacteria, Actinobacteria* and prevalence of *Acetobacteraceae* family and for yeasts, the *Saccharomycetaceae* and *Schizosaccharomycetaceae* families were dominant. The shotgun metagenomics examination of kombucha showed that the biofilm contains more bacterial species (*Acetobacter xylinum, A. indonesiensis, A. papaya*, *Komagataeibacter saccharivorans, Gluconobacter* spp., *Microbacterium* spp., *Bacillus licheniformis*), while the fermented liquid contains more yeast species such as *S. cerevisiae, S. ludwigi, Zygosaccharomyces* sp., *Brettanomyces (Dekkera) bruxellensis*, *Hanseniaspora valbyensis*, *H. opuntiae*, *Pichia fermentans*, and *Galactomyces geotrichum* [66,67,68,70,71]. Moreover, the comparison of shotgun metagenomics before and after the exposure of the samples in the cosmic space (for 1.5 years) determined disorganization of the KMC (kombucha mutualistic community) without possibility to recreate (during the 2.5 years) the initial population structure. At the same time, the microorganisms from the genus *Komagataeibacter, Pichia, Candida* and *Debaryomyces* were not affected, with no significant differences in community positions. The hopanoid lipids (membrane components involved in regulating membrane fluidity and stability of bacteria) have an important role in the surveillance of consortium’s species. The survived KMC species provided the population with the genetic heritage needed to resist long periods in extreme environments or extra-terrestrial conditions [72].

**Figure 7:**
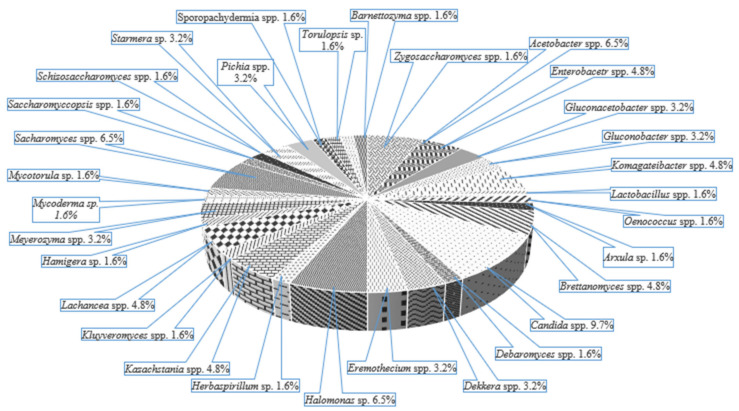
The microorganisms’ diversity in Kombucha (adapted from Soares et al. [73]).

Due to the interaction between many LAB, AAB and yeasts (acid-tolerant and osmophilic) in a mutualistic community, the abundance of species can be observed in Figure 7, kombucha being a probiotic drink and an effective source of probiotic microorganisms and probiotic candidates. *Pediococcus pentosaceus* and *P. acidilactici* originated from kombucha, contribute to the pleasant flavour of kombucha by forming SCFAs, furthermore, it produces bacteriocins and shows resistance to high bile salts concentration (0.5% *w*/*v*), antioxidant potential (56–58%), and high inhibitory activity of foodborne bacteria (*Salmonella enterica Typhimurium, Listeria monocytogenes, L. ivanovii, Bacillus cereus, Proteus hauseri*) and fungi (*Penicillium expansum* and *P. digitatum*) [74,75].

To improve the functionality of artisanal cultures or the utilization of the isolated strains, it is important to understand the complex interaction between species. Many strains of bacteria and yeast were isolated from the wild consortia of kombucha (*Hanseniaspora vineae, Torulaspora delbrueckii, Zygosaccharomyces kombuchaensis*) and kefir grains (*Leuc. mesenteroides* and *L. hilgardii, L. nagelii, L. hordei*), which were then studied and employed in different biotechnological applications (in the food preservation, formulation of alcohol-free beer or other fermented products, dextran production, or pharmaceutical industry, probiotic powders) [76,77,78,79]. Another way to develop new probiotics, symbiotic, symbiotic or postbiotic products is to formulate microorganisms consortia, especially the combination of LAB and yeasts (for example, the genus *Lactobacillus* ssp. and *Saccharomyces* spp.), to better control metabolites production and interactions between species [80,81,82]. The communication between strains is held by signalling molecules (quorum sensing) or by adhesion factors like proteins or polysaccharides (forming biofilm) [83]. The fermentation process of kombucha is a promising model system for multiple species cooperation research because of its cooperative and competitive interactions. These interactions are demonstrated by yeast strains which produce invertase for sucrose disruption then being used by the bacterial or other yeast strains. The competitive interaction can be observed in bacteria that produce cellulose thus protecting cells from outside competitors and retain desiccation [60].

Most of the experiments for understanding bacterial metabolism are based on studies of single strains or their growth in a mixed community. As a result, their growth can be stimulated or altered. A mutualism relationship was identified between a nitrogen formation yeast strain and a *Lactococcus lactis*, another important observation being related to the lactose that contributes to an easiest interaction between *L. lactis* and *Saccharomyces cerevisiae* [84]. Stadie et al. contrasted the growth of the co-cultivation vs. a pure culture of key representative water kefir isolates. The results showed that in a mixed fermentation the number of cells increases for both lactobacilli and yeasts than in separate cultivations. *Zygotorulaspora florentina* and *Saccharomyces* *cerevisiae* provide essential amino acids for *Lactobacillus* *nagelii* and vitamin B6 for *L. hordei*, but *Z. florentina* has a greater effect than *S. cerevisiae*. It was determined that the stimulation only occurs in co-cultivation, *Lactobacillus* species produced nutrients for yeast and the yeast, in turn, forming essential nutrients for lactobacilli. As a result of species metabolism and nutrient intake, the symbiotic interaction between yeasts and lactobacilli are present [83].

In the co-existing relationship between two dominant strains of *Lactobacillus kefiranofaciens* and a yeast strain of *Saccharomyces cerevisiae* from Tibetan kefir grains, it was determined that the cell size of *L. kefiranofaciens* is unlikely to be directly changed by the yeast metabolic activity in the kefir grains. The size of the cells from the pure culture of *L. kefiranofaciens* is rod-shaped (1.7–2.5 × 0.5–0.6 µm) while in Tibetan kefir grains two different sizes of rod-shaped cells were observed, respectively 3.0 µm in length on the outside surface of matrix and 10.0 µm in length in the inner parts of matrix [85].

Metabolites of one microorganism could induce the growth of another microbial strain in the kefir grain, because the metabolites that result from one strain are used as the carbon source for the other microorganism. The synergistic effect, commensalism or antagonism may appear when co-culture growth system is involved [86]. *S. cerevisiae* yeast strain by its catalase activity reduced the level of hydrogen peroxide (H_2_O_2_) and lactic acid produced by LAB. The presence of a high amount of hydrogen peroxide causes the self-growth-inhibition effect, as a result, LAB are inhibited by its metabolites. *S. cerevisiae* was able, as well, to develop synergistic relationships with *Leuc. mesenteroides* strains, the mixed co-culture increased the level of viable yeast cells up to 4 log CFU/mL, whereas only 2.6 log CFU/mL viable cells were determined for the single culture of *S. cerevisiae* [87].

The optimal combination between the microorganisms from kombucha (*Gluconacetobacter* spp.) and kefir obtained from milk kefir grains (10 isolates of LAB) was determined. LAB play an important role in the survival and metabolic activity of *Gluconacetobacter* spp. LAB strains influenced the metabolism of glucose into xylose, acetic acid; in this way they sustained the D-saccharic acid 1, 4 lactone (DSL) production from *Gluconacetobacter* spp. DSL and glucuronic acid are some functional metabolites found in Kombucha which have the capacity to inhibit glucosidase activity, thus decreasing the glycaemic response, or to minimize the carcinogenic effects of polycyclic aromatic hydrocarbons, amines, nitrosamines and fungal toxins [88,89].

## 4. The Postbiotic and Prebiotic Potential of Artisanal Starter Cultures in Regard to the Fermentation Substrate

Every microbial consortium has specific nutritional requirements that are directly influenced by their origin and availability. Nowadays, many unconventional and innovative substrates are studied in order to identify and assure the most suitable conditions to manage the controlled fermentation processes conducted by the artisanal cultures, thus increasing the functional characteristics of the fermented products [90,91]. Table 2 summarizes the information about the used fermentation substrates. Their diversity assures the complexity of the postbiotics’ and prebiotics’ composition.

Probiotic bacteria from milk kefir grains use the carbon- and nitrogen-based nutrients within the fermentation medium and produce metabolites such as organic acids or carbonyl compounds and esters. In this way, products with specific taste and aroma are obtained. In addition, the resulting amino acids, SCFAs, bacteriocins and enzymes are important bioactive compounds that improve the nutritional quality and microbiological safety of the final products [27,110]. Bioactive peptides with angiotensin-converting-enzyme (ACE) inhibitory properties and antioxidant activity were obtained by fermenting bovine colostrum with artisanal milk kefir grains by co-cultivation with the yeast strain *Candida lipolytica* [111]. The fermented product is considered a tribiotic fermented product that can represent a promising starting point for the further development of nutraceuticals and cosmeceuticals.

By co-cultivation of yeasts with LAB strains, the yield of bioactive peptides and amino acids can be maximized. The yeast strains can be growth factors for the LAB strains. These ones, in turn, reduce the pH of the fermentation medium for an optimal yeast growth. In the case of water kefir grains, *S. cerevisiae* was determined as a specific regulatory yeast. Metabolites’ production and kefir grain growth are also influenced by the characteristics of the water used and its concentration of calcium ions. The latter has an important role in growing the glucan chain and contributing to the optimal activity of glucansucrases. The low pH inhibited the production of glucansucrase, in this way harms kefir granules and forms ethanol production. The fermentation substrates for obtaining metabolites vary from various sugars and different types of fresh or dried fruits, vegetables and legumes made of raw substrates or extracts [112,113].

The cultivation of kefir grains in whey permeate-based medium increases the microbial growth kinetics and the antifungal activity of the fermented product. The use of cell-free supernatant (CFS) from the fermented liquid in poultry feed and bread making increases the fungal infection tolerance and the sensory properties [114].

The fermentation process contributes to lactose decreasing in milk due to its hydrolysis and acid production, lactose being the main carbon source for the microorganisms within the milk kefir grains. Additionally, the monosaccharides (glucose and galactose from the hydrolysis of lactose), oligosaccharides (sucrose, maltose, lactose), and prebiotics (kefiran formed by kefir grains’ microorganisms) are produced in the fermentation process [115]. Bioactive peptides could result after the proteins’ enzymatic hydrolysis during fermentation. This process is assured by the proteases and/or peptidases synthesized by the microorganisms from the kefir grains consortium. Some authors reported that water-soluble bioactive peptides were identified in fermented kefirs made of ewe or cow milk. The fermentation of these previously mentioned dairy substrates in controlled conditions, 5% (*w/v*) milk kefir grains inoculum, for maximum 48 h at 25 °C, highlighted that the ewe’s milk was more suitable for bioactive peptides’ production with antioxidant and antibacterial properties [116,117].

In Mexico, water kefir grains (Tibicos) are mainly used to produce Tepache (a fermented drink based on pineapple peel) [118]. The composition of the microbial species in the water kefir liquor and on the water kefir grains was the same and remained stable throughout the fermentation process. The sucrose from the fermentation medium was entirely transformed into fructose after 24 h of fermentation. The presence of sucrose determined the growth of water kefir grains and its microbial population, subsequently it influences the production of metabolites during the fermentation process. Ethanol, carbon dioxide, lactic acid, glycerol, and acetic acid, isoamyl acetate, ethyl hexanoate, ethyl octanoate, and ethyl decanoate were determined by GC, HPAEC-PAD (high-performance anion-exchange chromatography with pulsed amperometric detection), and HPLC [45].

The bacterial strains of the water kefir grains have specific physiological characteristics, namely their ability to produce homopolysaccharide matrix that contains dextran-based exopolysaccharides. The optimal condition for the production of dextran by the isolated, strains from water kefir grains, *Leuconostoc mesenteroides* and *Lactobacillus hilgardii* was 23–30 °C after 16–20 h [77,119]. A *G. liquefaciens* strain isolated from water kefir in Argentina produced 4-keto-D-arabonate, an enzyme that catalysed decarboxylation and dehydrogenation reactions [120].

Romero-Luna et al. [118] reported that a paraprobiotic strain of *L. paracasei* CT12 was isolated from water kefir grains. CFS of *L. paracasei* CT12 had antimicrobial properties against pathogenic bacteria (*E. coli*, *S. aureus*, *L. innocua*) and scavenging activities against mold strains (*Botrytis cinerea* and *Penicillium* spp.). The postbiotics produced by the microorganisms from the kefir grains interfere with pathogenic bacteria by adhesion of the intestinal mucosa, which in this way helps to improve the gut health [54].

Another interesting unconventional substrate used for water and milk kefir grains is the palm fresh sap obtained from *Palmyra Borassus aethiopum*. The palm fresh sap was inoculated with 4% (*w*/*v*) activated milk kefir grains and water kefir grains, the mixture being afterwards fermented in anaerobiosis for 48 h at 22 °C. The glucose concentration (after 48 h of fermentation) ranged between 1.61–8.45 g/L, fructose contents ranged from 0.93 up to 4.54 g/L, sucrose content decreased because of its transformation in the above-mentioned compounds. The obtained fermented product is useful for consumers with special nutritional needs, that may be dairy-allergic, lactose intolerants, vegetarians and vegans, particularly [121].

The utilization of kefir grains in sourdough bread increased the concentration of some volatile compounds such as 2-phenyl-ethanol, ethyl acetate, benzaldehyde, when the aroma profile was compared to that of a commercial sourdough bread. The kefir grains could be involved in cheese production and alcoholic fermentation for their potential utilization as starter cultures or preservatives [122]. Moreover, kefir grains are used as sourdough bread improvers regarding the sensorial attributes and self-life [123]. Including kefir grains into the bread making recipe seemed to reduce the staling rate, an improved spoilage resistance of sourdough bread being at the same time observed. Freeze-dried kefir grains represent a useful alternative for obtaining artisanal bakery products with a plain, mild dairy scent and a uniform crumb [57].

Kolakowski et al. [124] studied the behaviour of the milk kefir grains in stressful conditions, specifically at an incubation temperature of 18 °C together with a pathogenic strain of *Escherichia coli* previously inoculated in the pasteurized milk. After 24 h of incubation in these previously described conditions, it was observed that the microorganisms from the kefir grain consortium had an antipathogenic effect. Furthermore, this conclusion may suggest that the milk kefir grains employed in the fermentation process can be successfully used for other fermentation batches.

Some LAB strains are able to biosynthesize bioactive postbiotic compounds with antimicrobial and functional properties for foods such as exopolysaccharides (EPS), bioactive peptides, and SCFAs [125,126]. Additionally, the prebiotic EPS produced by an *L. paracasei* strain isolated from kefir had a significant impact on the activity and composition of the colonic microbiota in healthy children. The colonic fermentation of EPS increases the proportion of the microorganisms belonging to the genera *Victivallis, Acidaminococcus*, and *Comamonas*, as well as a substantial decrease in the proportion of enterobacteria. EPS produced by *L. paracasei* in milk can be considered bioactive compounds because they alter the intestinal microbiome (metabolized in vitro by faecal microbiota producing SCFAs) by enhancing the development of propionic and/or butyric acid, two metabolites that have been linked to a variety of health benefits [127].

Ten *Lactobacillus* spp. strains isolated from Indonesian milk kefir grains were capable of synthesizing compounds with varying degrees of inhibition against α-glucosidase, these lactobacilli’s metabolites being also responsible for the antioxidant activity. Among these ten strains, the isolates characterized by the highest glucosidase inhibition activity, and respectively by a superior antioxidant activity were *L. rhamnosus* and *L. kefiri*. Reconstituted skim milk is the most efficient medium for producing antioxidant compounds, being useful also to produce different kinds of fermented milk with strong antioxidant activity. The antioxidants’ production in skimmed milk is linked to production of peptides by the cultures’ proteolytic activity, which primarily affects casein. The identification of small peptides’ fractions (<3 kDa) revealed high antioxidant activity. Sequences with well-known antioxidant properties were found in some peptides [128]. The bioactive peptides gained the scientists’ interest due to their immunomodulating, antioxidant, and antimicrobial activities and their usefulness as bioactive ingredients in functional food products. These peptides can be obtained after the fermentation process of milk with milk kefir grains thanks to the action of the endogenous proteinases, thus, the microbiological degradation plays an essential role in this process. Ebner et al. [129], reported that an increase regarding the bioactive peptides sequences of α -S_1_-casein, α-S_2_-casein, β-casein, and κ-casein were identified in kefir obtained with milk kefir grains.

The traditional substrate made of green and/or black tea for the kombucha’s fermentation was substituted by 10% (*v*/*v*) guava fruit (*Psidium guajava*) extract and subjected to a new fermentation for 7 days at 25–30 °C. The supernatant showed antibacterial activity which was confirmed by the high inhibition zones against *Pseudomonas* spp. (Z.I. = 29.01 ± 0.1 mm), *Klebsiella* spp. (Z.I. = 26.98 ± 0.09 mm) and *Staphylococcus* spp. (Z.I. = 25.01 ± 0.07 mm). The antifungal and antibacterial activities may be due to the presence of the metabolites, polyphenols, and flavonoids. These results were supported by a detailed HPLC analysis of the kombucha’s broth, showing that the gallic acid, catechin, guaiacol and coumaric acid were identified as the major bioactives [105].

The fermentation broth of the jujube kernel with kombucha SCOBY contained more functional compounds and it was characterized by an enhanced antioxidant activity compared to the fermentation medium without kombucha. Furthermore, microbiota’s bioconversion capacity contributes to the formation of jujuboside B (known for its utilization in insomnia and anxiety treatment), a compound that was found in a concentration of 0.011 g/L after 10 days of fermentation, the presence of jujuboside B indicating the benefit of the mixed fermentation. Kombucha SCOBY can be used in the fermentation process of useful plants to improve their medicinal effects [107].

The kombucha consortium is more efficient in the degradation of aflatoxin B1 in black tea compared to the isolated kombucha strains. This aspect can be explained by the fact that, through the fermentation process, lactic acid bacteria opens up the aflatoxin B1 lactone ring resulting in its complete detoxification [130]. The kombucha culture reduced the level of AFB1 in black tea with high efficiency (97%). The biodegradation percentage for the isolated strains was 59% for *Pichia occidentalis* and 39% for *Hanseniaspora opuntiae*. The colorimetric 3-(4,5-dimethylthiazolyl-2)- 2,5-diphenyltetrazolium bromide (MTT) test on Hep2 cells and the brine shrimp (*Artemia salina*) lethality assay revealed a substantial increase in toxicity of the by-products. After biodegradation by kombucha culture, toxicity assessment of the by-products using MTT test on Hep2 cells and the brine shrimp (*A. salina*) lethality assay revealed a substantial decrease in AFB1. As such, it results in a novel way to reduce AFB1 toxicity by natural kombucha that can be used in the food and feed industry [131].

The bioactive compounds (polyphenols, gluconic acid, glucuronic acid, lactic acid, vitamins) are mostly present in fermented sugared black tea by kombucha [132]. For example, D-saccharic acid-1,4-lactone (DSL) are found just in the fermented product. The concentration of this compound increased with the extension of the time of fermentation and reached a concentration of 2.24 ± 0.1 g/L after 21 days. Simultaneously, the level of polyphenols and flavonoids increased respectively by 54% and 24% [133].

The symbiotic community of kombucha include LAB and bifidobacteria, their synergism produces many postbiotics such as organic acids, polyphenolic compounds, and water-soluble vitamins. Especially, the complex content of B-group vitamins contributes to the equilibration of the blood pH and the lactic acid concentration. It was demonstrated that the supplementation of wheat-based probiotic diets with 20% of kombucha fermented beverage improved the broiler’s performance and growth. Furthermore, the probiotic diet helps to increase the villus (vascular projections that increase the surface area for food absorption and adding digestive secretions) height/crypt and depth ratio of the intestinal mucosa [134]. The key metabolites including acetic, lactic, malic, and succinic acid are formed in the fermentation phase of kombucha due to yeast–bacterium interaction [68]. Under optimal fermentation conditions, gluconic acid is one of the health-promoting metabolites present in kombucha that fermented sucrose and black tea. Gluconic acid dehydrogenase and 2-ketogluconate dehydrogenase, respectively, transform gluconic acid to 2-ketogluconate and then to 2,5-diketogluconic acid during oxidation. Antioxidants production is largely attributed to the bioactive compounds found in fermented foods and drinks [103].

**Figure 8:**
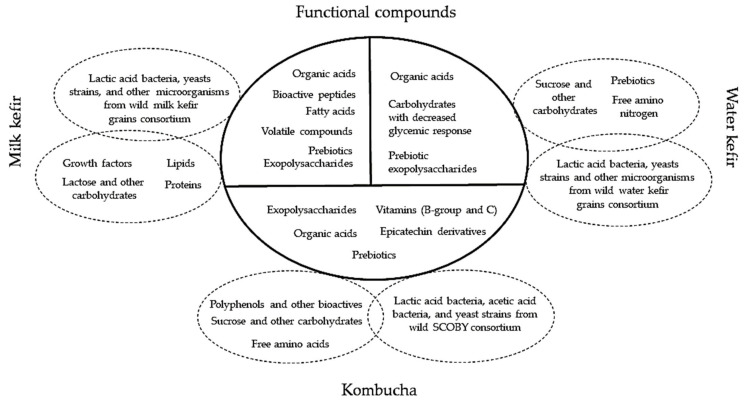
Functional metabolites within the milk and water kefir, and respectively kombucha beverages.

Milk and water kefir beverages, as well as kombucha, are characterized by various functional compounds that were summarized in Figure 8. Therefore, these metabolites can have positive health effects for the consumers. Consequently, the mechanisms responsible for these beneficial effects are intensively studied in the literature. The wild consortia of milk and water kefir grains, and respectively kombucha, can be used as starters by co-cultivation on optimized fermentation substrates in order to maximize the functional traits of the final product.

Milk kefir grains contain considerable levels of proteins, galactooligosaccharides, and extracellular enzymes. Lactose and proteins are biotransformed during fermentation and produce carbon dioxide, lactic acid, acetaldehyde, acetoin, minor quantities of ethanol and 50 distinct aroma compounds [135]. By comparison, water kefir grains ferment sucrose, glucose, fructose, and/or mannitol and produce lactic acid, acetic acid, ethanol, carbon dioxide, volatile compound- methyl esters and other 30 distinct chemicals [136]. Besides kefir grains, the fermentation process of kombucha SCOBY produces many other bioactive compounds such as polyphenols, flavonoids, sucrose, glucose and fructose, vitamins B1, B2, B6, B12, C, amino acids, purines, pigments, lipids, proteins, some hydrolytic enzymes, acetic, gluconic, glucuronic, citric, succinic, malic, tartaric, malonic and oxalic, L-lactic, D-sugar, pyruvic, and usnic acids [137,138,139]. The fermentation of *Medusomyces gysevii* with *Vaccinium myrtillus*, *Callisia fragrans* (kombucha SCOBY) produces vitamins, especially vitamin C and vitamin P (rutoside), after 10 days of fermentation their presence are the highest [109].

## 5. Obtaining Paraprobiotics by Cell Disruption and Their Applications

**Figure 9:**
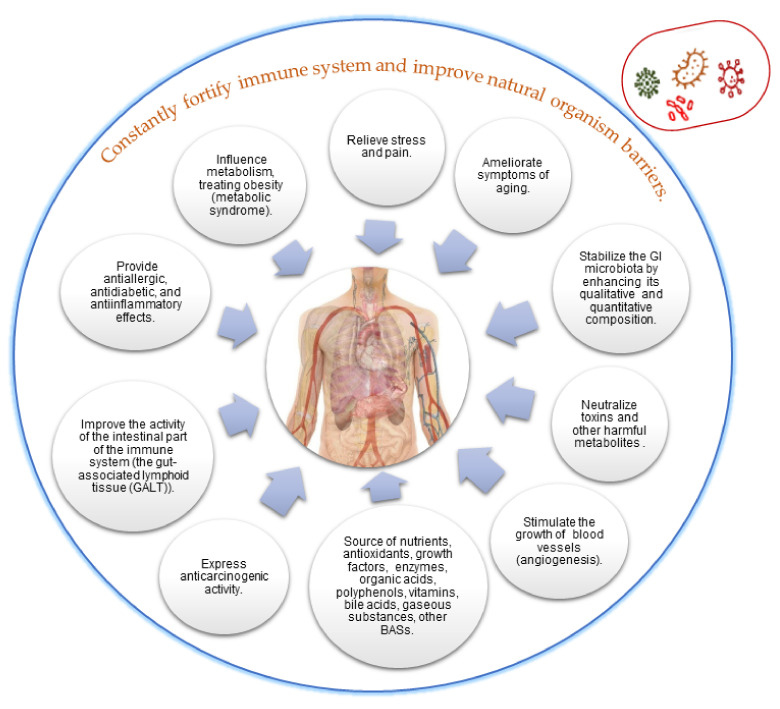
Health-promoting effects of metabiotics on the host organism (adapted from Oleskin at al. [35]).

The metabiotics are considered more suitable and convenient for long-term storage of the functional products. Metabiotics have a clear target and mode of action (Figure 9), being easier to dose or control their safety in comparison with other traditional probiotics [140]. The strengths of metabiotics are: their high bioavailability because metabiotic substances reach the colon by 95–97% of them remain unchanged (they do not interfere with the human’s microbiome); they are active and begin to work immediately after administration, being mainly responsible for the positive effects on the digestive tract [141]. Furthermore, metabiotics comprise a wide spectrum of functional compounds with various beneficial effects for the consumers.

Overall, a wide range of factors, such as the consortium composition, the types of predominant bacteria and yeast strains, the fermentation time, the composition of the fermentation substrate were identified to have a significant impact on the postbiotics, probiotics, prebiotics and paraprobiotics production. The effectiveness of cell lysates, CFS, non-viable cells, EPS, teichoic and lipoteichoic acids, lipopolysaccharides, peptidoglycans originated from LAB strains (*Lactobacillus* spp., *Bifidobacterium* spp.) and yeasts (*Saccharomyces* spp.). The effectiveness is influenced by the dose and time of administration in order to reach satisfactory effects regarding the amelioration of some diseases [142].

Metabiotics can be obtained naturally after the fermentation processes. As such, *L. satsumensis* was able to metabolize glucose and fructose, producing glucan- or fructan- based EPS; the strains of *O. kitaharae*, *O. oeni*, *G. oxydans*, *Acetobacter* spp., *G. cerinus*, *K. intermedius*, *K. saccharivorans* were involved in the malolactic fermentation, their ability to oxidize ethanol into acetic acid or sugars and sugar alcohols into the corresponding organic acids was determined; *B. bruxellensis, S. cerevisiae, Lachancea fermentati* strains were able to produce hydrolytic enzymes involved in sucrose metabolism, these previously mentioned yeast strains remained active in stressful conditions, namely starving or low concentrations of alcohol [101].

Techniques for metabiotic obtaining are very different, depending on the desired compound to be obtained and its future destination. For example, metabiotic subclasses like paraprobiotic, postbiotic or prebiotic need advanced techniques for the disruption, inactivation of cells or extraction of bioactive compounds process that requires analysis to prove the health promoting effects of compounds. The most frequent techniques used for cells’ disruption are the following: mechanical destruction; frequent freezing and thawing; enzymatic lysis of the microbial cell walls, membranes or intracellular structures; microwave, ionizing, and ultraviolet radiations; supercritical water extraction; high hydrostatic pressure; ultrasound treatment; cell damaging by acidic or basal chemical solutions; centrifugation; dialysis; the use of ion exchange resins; ultrafiltration techniques applied for the whole or partially destroyed cells’ suspensions; inactivation of the microorganisms from a suspension and extraction of cells’ components and metabolites using CO_2_ [143]. Considering the nature of the treatment applied for cells’ disruption, there can be identified two main groups: (i) disruption procedures that require mechanical force—high-pressure homogenization, hydrodynamic cavitation, mechanical agitation (bead, mill, mortar and pestle, bead beating) and (ii) physico-chemical disruption techniques (extreme temperatures and pH values, osmotic shock, desiccation, gas decompression, sonication, hydrodynamic cavitation, detergents, solvents, antibiotics, chelating agents, chaotropes, exogeneous cell wall-lytic enzymes, autolysis and induced lysis, cell walls’ inhibitors). After the disruption of the cellular membranes, soluble intercellular proteins, plasmid DNA and polymeric compounds are released [144].

The various techniques used for obtaining metabiotic products offer, at the same time, numerous possibilities to study their chemical nature and functions for a better understanding and for their proper utilization in the future. Some authors reported and described chromatographic (GC- or LC-MS), spectroscopic (NMR) or gel electrophoresis-based protocols that were successfully used for the analysis of metabiotics.

The main compounds resulting after the inactivation or disruption of cell membranes of LAB cells are peptidoglycans, polymeric glycans, EPS, DNA fragments and lipoteichoic acids. The inactivated cells of LAB can be utilised for functional dairy products manufacture. Therefore, it was reported that the presence of EPS, killed cells (at 121 °C for 15 min) and cells’ components of *L. acidophilus* and *Bifidobacterium lactis* in a yogurt contributed, on one hand, to the syneresis minimization and, on the other hand, increased the water holding capacity and the apparent viscosity [145].

For the artisanal cultures, there are three basic structures as follows: kefiran is specific to milk kefir grains, dextran to water kefir grains, and cellulose fibrils to kombucha which degradation/denaturation gives useful compounds. Firstly, from kefir grains it can be extracted a heteropolysaccharide, named kefiran with molecular weight ranging between 2.4 × 10^6^ Da and 1.5 × 10^7^ Da. Kefiran is recognized as a GRAS (generally recognized as safe) compound with rheological and health-related properties, such as antibacterial, antifungal, and antitumoral, also upon enzymatic degradation of kefiran are obtained galactose and glucose. Kefiran and its derivatives can be used for the production of food packaging materials and encapsulation materials, as a drug carrier and prebiotic, emulsifier or gelling agent in functional foods [146,147]. Secondly, from the water kefir grain it can be extracted a very large polysaccharide—dextran—formed by α-1,6 glycosidic linkage along with α-1,3 branching with molecular weight starting from 4 × 10^5^ Da up to 10 × 10^6^ Da [148]. Usually, its degradation is carried out under the ionizing radiations on aqueous dextran solutions. Specifically, the T-irradiation ranging between 0–0.32 MGy was applied on H_2_SO_4_ acidified aqueous to degrade the macromolecules of dextran into hydrolysis’ products made with lower molecular weight fractions [149]. Low molecular weight dextran (LMWD) is suitable for pharmaceutical applications and is frequently used as a drug carrier. LMWD can be obtained after prolonged enzymatic hydrolysis of dextran’s macromolecules using citrate phosphate buffer (0.1 M, pH 4.5) and dextranase (250 U) at 50 °C for 72 h, followed by filtration (molecular weight cut-off filter membranes method) [150].

Finally, the extracellular polysaccharides produced by *Gluconobacter* spp. and *Acetobacter* spp. formed the cellulose fibrils of kombucha biofilm. The cellulose that results from kombucha’s fermentation can be used in the nanocomposites’ industry [151]. Moreover, hydrogels are made from the kombucha’s biofilm. Briefly, the process starts with conventional sterilization of kombucha’s biofilm in glycerol solution (6 and 12% *w*/*v*) the kombucha’s biofilm being previously frozen. Afterwards, the sterilized mixture is combined with the solution of stearic acid solution in ethanol (2.5% *w/v*). The kombucha-based hydrogel production process ends with a stream of hot air (70–75 °C) [152]. The bacterial cellulose of kombucha is rich in glycosaminoglycans, glycoproteins, and glycolipids and it is used as a polymer to create scaffolds for tissue engineering, cell transplantation, and possible cure for diabetes. This biofilm was tested by its seeding with insulin-secreting beta cell derived line (INS-1 cells) and it was observed that biofilm facilitates the beneficial morphological changes of islet cells [153].

Furthermore, the extract of kombucha as a low-cost and easy-to-obtain alternative treatment that enhances the immune system, overcomes some respiratory problems or intestinal parasites, and is efficient as well in maintaining the physiological condition of the animals, which is expressed in weight gain and lower production costs [154]. Kombucha is efficient in curing skin and muscular lesions due to its mild swelling, good cell proliferation, and well-organized structure that serves as a suitable support for collagen and elastin fibres that are involved in the tissues’ healing processes [155]. In comparison with the unfermented tea, kombucha fermented beverage has anti-inflammatory properties, thanks to the inhibition of certain inflammatory enzymes. It was discovered that kombucha G had the highest anti-inflammatory efficiency after fermentation, with a 44.5% inhibition rate of the inflammatory enzymes, compared to the consortium (H) with a 28% inhibition ratio in the ethyl acetate-based extracts. The anti-inflammatory ability of kombucha consortia against the 15-lipoxygenase enzyme was supported by these findings [70].

## 6. Conclusions and Future Perspectives

The current preoccupations in food biotechnology enable scientists to use symbiotic wild cultures of microorganisms from consortia to produce innovative, healthy, and functional food products and ingredients rich in metabiotics. The latter can be obtained using artisanal consortia and advanced techniques. The microorganisms within the wild artisanal cultures develop stable and mutual relationships useful for their survival and development. Hence, such consortia contain multiple probiotic strains, characterized by a complex metabolic activity and physiology, being able to produce various types and considerable amounts of postbiotics and paraprobiotics that must be further studied and exploited for metabiotics and functional food production with enhanced overall quality, stability, and safety assurance. Metabiotic production by exploiting the artisanal cultures and the unconventional substrates is a promising challenge in food and feed production, also including ingredients and nutraceuticals. In this context, it could be possible to formulate planned metabiotic ingredients, adapted to the specific type of intestinal microbiocenosis disorder with a great impact on immunity of organisms and its vital activity.

## Figures and Tables

**Table 1:** The novel taxonomy of *Lactobacillus* spp.

Genus	Old Taxonomy	New Taxonomy
*Lactobacillus*	*Lactobacillus casei*	*Lacticaseibacillus casei*
	*Lactobacillus rhamnosus*	*Lacticaseibacillus rhamnosus*
	*Lactobacillus brevis*	*Levilactobacillus brevis*
	*Lactobacillus hordei*	*Liquorilactobacillus hordei*
	*Lactobacillus fermentum*	*Limosilactobacillus fermentum*
	*Lactobacillus paracasei*	*Lacticaseibacillus paracasei*
	*Lactobacillus plantarum*	*Lactiplantibacillus plantarum*
	*Lactobacillus reuteri*	*Limosilactobacillus reuteri*
	*Lactobacillus acidophilus*	*Lactobacillus acidophilus*
	*Lactobacillus curvatus*	*Latilactobacillus curvatus*
	*Lactobacillus delbrueckii*	*Lactobacillus delbrueckii*
	*Lactobacillus sanfranciscensis*	*Fructilactobacillus sanfranciscensis*
	*Lactobacillus helveticus*	*Lactobacillus helveticus*
	*Lactobacillus sakei*	*Latilactobacillus sakei*

**Table 2:** Unconventional substrates used for the fermentation with artisanal cultures.

Artisanal Culture	Raw Material/Substrate of Fermentation	Benefits/Findings	References
Milk kefir grains	Sugarcane concentrate	Alternatives for the development of non-dairy foods, prevention of gastrointestinal diseases and strengthening the immune system	[92]
	Whole and skim milk	Improved plasma and hepatic lipide profile in rats	[93]
	Non-fat milk + sweet whey powder	Bacterial proteolytic activities contribute to the beneficial effects of whey fermented with kefir grains	[94]
	Goat milk	β-casein with an ACE inhibitor and antioxidant activity	[55]
	Bovine milk	Detecting ketones, esters, acetates, alcohols, and acids	[95]
	Milk with 2.8% fat	Probiotic beverage	[89]
	Bovine colostrum	Fermented product with antimicrobial properties	[39]
Water kefir grains	Tomato seed protein isolate	Protein-rich isolate from the tomato seed meal was converted to antioxidant hydrolysates	[96]
	Soy whey	Novel bioactive beverage with high functional potential	[97]
	Soybean milk and black bean milk	Good alternative substrates for kefir yogurt production with probiotic potential	[98]
	Tap water + cane sugar + fig extract	Detection of Bifidobacterium psychraerophilum/crudilactis	[45]
	Osmotically dehydrated (OD) pineapple	Production of a potential symbiotic beverage of potentially high added value	[99]
	Tap water + figs + lemon	Dextrans obtaining	[44]
	Apple, quince, grape, kiwifruit, prickly pear, and pomegranate juice	Developing fruit-based kefir-like beverages with high added value and functional properties	[100]
	Red pitaya and apple pulp	Producing a new functional beverage	[101]
	Coconut Water Agar (CWA) and CWA supplemented with yeast extract (CWAY) especially for strains isolation	Two alternative and salutary media for culture of kefir strains	[102]
Kombucha	Fresh and ripen soursop (Annona muricata L.) fruits	High potential to improve the quality, metabolites content, biological activity, and the Halal status of soursop kombucha	[103]
	Black tea + sugar	Invertase in kombucha tea increase the nutritional value of fermented product for diabetes patients	[104]
	Green/black tea + guava juice	Antimicrobial activity against human pathogenic bacterial strains and pathogenic fungi	[105]
	Lactose and lactose-free milk	Due to active microflora and organic acids, have a confirmed positive effect on the human body	[106]
	Jujube kernel	Obtaining of functional beverages and jujuboside B	[107]
	Unbleached wheat flour	Encapsulated kombucha-like sourdough starter for production of functional sourdough bread with extended shelf life and improved quality	[108]
	Back tea with total replacement or in combination with Melissa officinalis, Quercus robur, Vaccinium myrtillus, Callisia fragrans	Ascorbic acid and rutin obtaining	[109]
